# First record of *Achalinus hunanensis* Ma, Shi, Xiang, Shu & Jiang, 2023 (Serpentes, Xenodermidae) in Guangxi, China, with supplementary morphological information

**DOI:** 10.3897/BDJ.13.e168622

**Published:** 2025-11-27

**Authors:** Yanyuan Xie, Yuhui Li, Zhengjun Wu, Zening Chen

**Affiliations:** 1 Key Laboratory of Ecology of Rare and Endangered Species and Environmental Protection (Guangxi Normal University), Ministry of Education, Guilin, China Key Laboratory of Ecology of Rare and Endangered Species and Environmental Protection (Guangxi Normal University), Ministry of Education Guilin China; 2 Guangxi Key Laboratory of Rare and Endangered Animal Ecology, Guangxi Normal University, Guilin, China Guangxi Key Laboratory of Rare and Endangered Animal Ecology, Guangxi Normal University Guilin China; 3 College of Life Sciences, Guangxi Normal University, Guilin, China, Guilin, China College of Life Sciences, Guangxi Normal University, Guilin, China Guilin China; 4 Authors contributed equally to this work, Guilin, China Authors contributed equally to this work Guilin China

**Keywords:** *Achalinus hunanensis*, morphology, phylogenetics, taxonomy, Guangxi

## Abstract

**Background:**

The family Xenodermidae comprises six recognised genera: *Xenodermus *Reinhardt, 1836; *Achalinus *Peters, 1869; *Stoliczkia *Jerdon, 1870; *Fimbrios *Smith, 1921;* Parafimbrios *Teynié, David, Lottier, Le, Vidal & Nguyen, 2015; and *Paraxenodermus *Deepak, Lalronunga, Lalhmingliani, Das, Narayanan, Das & Gower, 2021. Amongst these, *Achalinus *Peters, 1869, is the most species-rich taxon. It is widely distributed across eastern and south-eastern Asia, ranging from northern Vietnam to south-western China and extending partially into Japan.

*Achalinus hunanensis* Ma, Shi, Xiang, Shu & Jiang, 2023 was originally described, based on two male specimens collected from Huaihua City and Changsha City, Hunan Province, China. Subsequent surveys also recorded this species in Dushan County, Guizhou Province, marking its first known occurrence there and providing the first description of female specimens. During our herpetological surveys in 2024, three *Achalinus *specimens were collected from Guangxi Zhuang Autonomous Region, China. Due to severe desiccation, DNA extraction was unsuccessful for specimen GXNU2024090120. However, we analysed mitochondrial DNA sequence data from the remaining two specimens and compared them with all known congeners. Both the morphological and molecular evidence strongly support the identification of the specimens from Jiuwanshan National Nature Reserve as *A*.* hunanensis*. Additional morphological data are provided here, based on the examination of three newly-collected individuals.

**New information:**

In this study, we provide a detailed morphological description of *A. hunanensis,* based on newly-collected specimens. We also present an updated species diagnosis incorporating additional morphological characters and revise the known distribution of *A. hunanensis.*

## Introduction

The genus *Achalinus* Peters, 1869, which currently comprises 28 recognised species, represents the most diverse genus within the family Xenodermidae and is distributed across northern Vietnam, China and Japan (Uetz et al. 2025). With the expansion of extensive field surveys and advances in molecular techniques, the previously underestimated biodiversity within the genus *Achalinus* is being increasingly revealed. Since 2019, more than 20 new species have been described within this genus (Peters (1869), Boulenger (1888), Boulenger (1908), Pope (1935), Bourret (1937), Zong and Ma (1983), Ota and Toyama (1989), Zhao et al. (1998), Wang et al. 2019, Ziegler et al. 2019, Li et al. 2020, Miller et al. 2020, Huang et al. 2021, Hou et al. 2021, Pham et al. 2023, Li et al. 2021, Yang et al. 2022, Ma et al. 2023a, Ma et al. 2023c, Xu et al. 2023, Yang et al. 2023, Zhang et al. 2023, Zhang et al. 2023, Li et al. 2024, Huang et al. 2024, Xu et al. 2024). *Achalinus* are small-sized snakes and characterised by fossorial and secretive habits, typically inhabiting moist microhabitats, such as forest floor leaf litter or beneath decaying logs in mountainous regions. Their activity patterns show strong environmental dependence, with surface activity primarily occurring at night or under rainy conditions. The combination of limited mobility and specialised habitat preferences makes field collection particularly challenging, leading to an extreme scarcity of specimens available for study. The* A*.* hunanensis *population identified in Guangxi shows notable morphological divergence from previously reported populations; amongst the three adult specimens examined, two females exhibiting a distinct occipital patch extending across the head and abdomen — a feature absent in males. Given the current scarcity and distinctiveness of available specimens, a systematic and supplementary morphological characterisation of this species is urgently needed.

The Hunan odd-scaled snake, *Achalinus hunanensis* Ma, Shi, Xiang, Shu & Jiang, 2023, was originally described, based on two male specimens. Subsequently, Xu et al. (2025) reported a female specimen from Dushan County, Guizhou Province, thereby extending the known distribution range of this species. Currently, *A. hunanensis* is documented from Huaihua City and Changsha City in Hunan Province and Dushan County in Guizhou Province, China. It can be distinguished from its congeners primarily by the following combination of morphological characters: (1) dorsal scales strongly keeled, 23 rows throughout the body, the outermost row strongly keeled and substantially enlarged; (2) tail relatively short, TAL/TL ratio 0.22-0.24 in males and 0.17 in females; (3) maxillary teeth 23; (4) length of suture between internasals substantially longer than that between prefrontals, LSBI/LSBP ratio 2.0-2.1; (5) one loreal, subrectangular; (6) six supralabials, the fourth and fifth in contact with the eye; (7) the two anterior temporals in contact with eye; (8) ventrals 163-168 in males and about 169 in females; (9) subcaudals 69-72 in males and about 53 in females, not paired （Xu et al. 2025）.

**Figure 1.**
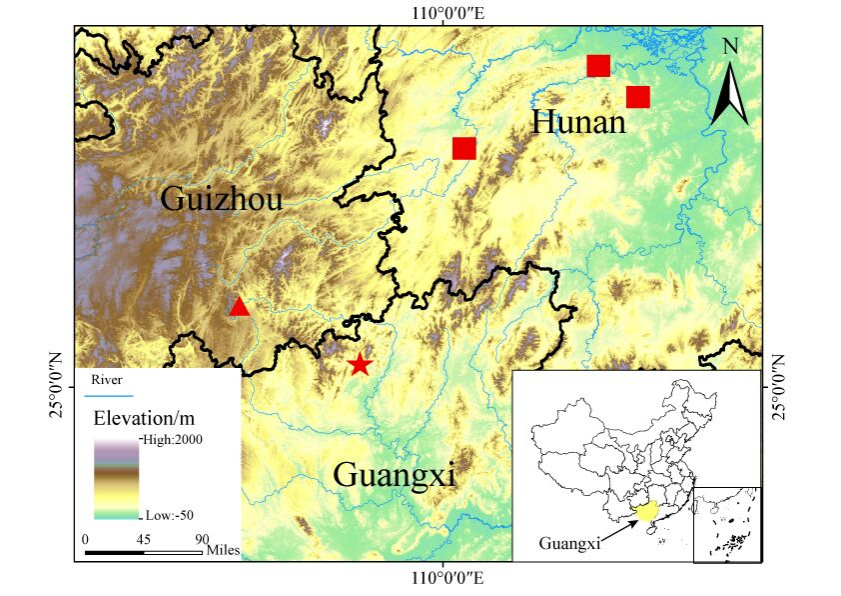
Distribution of *Achalinus hunanensis*. The squares indicate the type localities in Hunan, the triangle representing locality in Guizhou and the star marking the sampling site in the present study. Elevation data source: CGIAR-CSI SRTM v.4.1.

In recent surveys in Guangxi, China, three *Achalinus* specimens were found in Jiuwanshan National Nature Reserve (Fig. 1). Molecular analyses revealed that the specimens from Rongshui Miao Autonomous County first clustered with the specimens from Guizhou Province and subsequently formed a clade with the type series of *A. hunanensis.* Morphological examinations further supported these findings. Therefore, we provide supplementary descriptions of the new specimens, an expanded morphological diagnosis and new distribution records for Guangxi.

## Materials and methods

**Sampling**

Three* Achalinus *specimens were collected in 2024 from the Jiuwanshan National Nature Reserve in Rongshui Miao Autonomous County, Liuzhou, Guangxi, China Fig. 1. The specimens include one adult female GXNU20240905009 (25.21649174°N, 108.68016354°E, 891 m a.s.l.), one adult male GXNU2024102112 (25.22070854°N, 108.6763514°E, 983 m a.s.l.) and one dried roadkill specimen GXNU2024090120 (25.43329212°N, 109.16085809°E, 1281 m a.s.l.), all collected by Li Ding. Specimens were fixed in 80% ethanol and deposited at the School of Life Sciences, Guangxi Normal University. All sampling complied with China's Wild Animal Protection Law and sex was determined via gonadal dissection.

**Molecular phylogen** **etic analysis**

Genomic DNA was extracted from liver tissue using the Hi-Pure Animal Genomic DNA Kit. The partial mitochondrial cytochrome c oxidase 1 (COI) gene was amplified with primers RepCOIF and RepCOIR (Nagy et al. 2012). The Polymerase chain reaction (PCR) products were sequenced by Beijing Qingke New Industry Biotechnology Co., Ltd. For phylogenetic analysis, 35 sequences (Table 1) were selected, including 33 sequences obtained from GenBank. These included 30 sequences from 26 *Achalinus* species and three outgroup sequences from *Fimbrios klossi*, *Parafimbrios lao* and *Xenodermus javanicus *(Luu et al. 2020, Ha et al. 2022).

All sequences were aligned with other retrieved sequences from the same gene loci using MEGA 11 (Tamura et al. 2021). Uncorrected pairwise distances (p-distance) were then calculated in MEGA11. Phylogenetic trees were constructed, based on COI gene using Maximum Likelihood (ML) and Bayesian Inference (BI). For the ML analysis, RAxML v.8.2.4 (Stamatakis 2014) was employed with the GTR+GAMMA substitution model and nodal support was assessed through 1000 bootstrap replicates with the rapid bootstrap algorithm. Bayesian analysis was conducted using MrBayes 3.2 (Ronquist et al. 2012) with nucleotide substitution models selected by PartitionFinder 2.1.1 (Lanfear et al. 2012, Lanfear et al. 2017), based on the Bayesian information criterion (BIC), which identified K80+I+G, HKY+I and GTR+G as optimal models for different partitions.The Markov Chain Monte Carlo (MCMC) analysis ran for 20 million generations, sampling every 1000 generations. Meanwhile, uncorrected pairwise genetic distances amongst *Achalinus* species were calculated in MEGA 11 after excluding outgroups (Kumar et al. 2018) (Table 1).

**Morphological examination**

Morphometric data collection, scale enumeration and standardisation of morphological abbreviations all followed the methodologies outlined by Zhao (2006) and Xu et al. (2025). Three key measurements were taken using the Yong Guang Stainless Ruler (00722098) to the nearest mm: snout-vent length (**SVL**), tail length (**TAL**) and total length (**TL**). Other measurements were taken with Deli digital calipers (DL3945) to the nearest 0.1 mm: head length (**HL**, length from the tip of snout to the posterior margin of mandible; head width (**HW**, width from the widest part of the head in dorsal view); eye diameter (**ED**, diameter from the most anterior corner of the eye to the most posterior corner); loreal height (**LorH**, height from the highest part to the lowest part of the loreal in lateral view); loreal length **(LorL**, length from the most anterior loreal to the most posterior loreal in lateral view); length of the suture between internasals (**LSBI**); length of the suture between prefrontals (**LSBP**).

The scale features and their abbreviations are as follows: loreals (**Lor**); supralabials (**SL**); infralabials (**IL**); number of chin shield pairs (**Chins**); infralabials touching the first pair of chin shields (**IFL-1st Chin**); temporals (**TEMP**); supraoculars (**SPO**); dorsal scale rows (**DSR**) (counted at one-head-length behind the head, at midbody and at one-head-length before the anal), number of anterior temporals that touch the eye (**aTEM-Eye**) (head bilateral scale counts are given as left/right), ventral scales (**VS**), cloacal plate (**CP**), subcaudal (**SC**).

## Taxon treatments

### Achalinus hunanensis

Ma, Shi, Xiang, Shu & Jiang, 2023

30DD1015-7CB5-5FA9-A841-DD08A0F4E51A

https://doi.org/10.15468/eh9asg

#### Materials

- **Type status:** Other material. **Occurrence:** catalogNumber: GXNU20240905009; individualCount: 1; sex: female; occurrenceID: 182A6960-3B27-56D2-B35E-40C1433E1BC4; **Taxon:** scientificName: *Achalinus hunanensis*; order: Squamata; family: Xenodermidae; genus: *Achalinus*; **Location:** country: China; stateProvince: Guangxi; county: Rongshui Miao Autonomous; verbatimElevation: 891 m; verbatimCoordinates: 25°12.99'N 108°40.81'E; **Event:** eventDate: 28-08-2024
- **Type status:** Other material. **Occurrence:** catalogNumber: GXNU2024090120; individualCount: 1; sex: female; occurrenceID: DCB238DB-23AA-5ABC-AF22-68A659E8E4AB; **Taxon:** scientificName: *Achalinus hunanensis*; order: Squamata; family: Xenodermidae; genus: *Achalinus*; **Location:** country: China; stateProvince:  Guangxi; county: Rongshui Miao Autonomous; verbatimElevation: 1281 m; verbatimCoordinates: 25°26.00'N 109°09.65'E; **Event:** eventDate: 30-08-2024
- **Type status:** Other material. **Occurrence:** catalogNumber: GXNU2024102112; individualCount: 1; sex: male; occurrenceID: E152E3EE-B346-50C1-83C7-5DDB57E948EB; **Taxon:** scientificName: *Achalinus hunanensis*; order: Squamata; family: Xenodermidae; genus: *Achalinus*; **Location:** country: China; stateProvince:  Guangxi; county: Rongshui Miao Autonomous; verbatimElevation: 983 m; verbatimCoordinates: 25°13.24'N 108°40.58'E; **Event:** eventDate: 15-10-2024

#### Description

The description is based on the specimens from Guangxi. The measurements and scalation characters of *A. hunanensis* are listed in Table 2. Body slender, cylindrical; head slightly distinct from neck; eye small; triangular, slightly visible from above; length of the suture between the internasal substantially longer than that between prefrontal, with LSBI/LSBP ratios > 1; nostril positioned in anterior part of nasal, deeply concave inwards; prefrontals paired; frontal pentagonal, pointed to the rear, slightly wider than high, much shorter than the parietals; loreal one, subrectangular, with LorH/LorL ratios < 1; supraocularone, Irregularly hexagonal; TEMP 7/7, arranged in three rows (2+2+3 on both sides), the anterior two contact the eye; the upper one smaller, irregularly quadrilateral, the lower one elongated; six supralabials, the fourth and fifth contacting the eye, the last one much elongated; two pairs of chin shields, the anterior pairs almost equal to the posterior pairs (GXNU2024102112 left two scales, right three scales and the second pair on the right side divided into two smaller scales), followed by preventrals; one mental, followed by 5/5 infralabials, the first one contacting with others after the mental and before the 1^st^ chin-shields, 1^st^-3^rd^ touching the first pair of chin-shields.

Dorsal scales strongly keeled, lanceolate, 23 rows throughout the body, the outermost row strongly keeled and substantially enlarged. Female GXNU20240905009: VS 173, SC 61; Male GXNU2024102112: VS 163, SC 71; anal entire; subcaudals unpaired.

**Figure 2.**
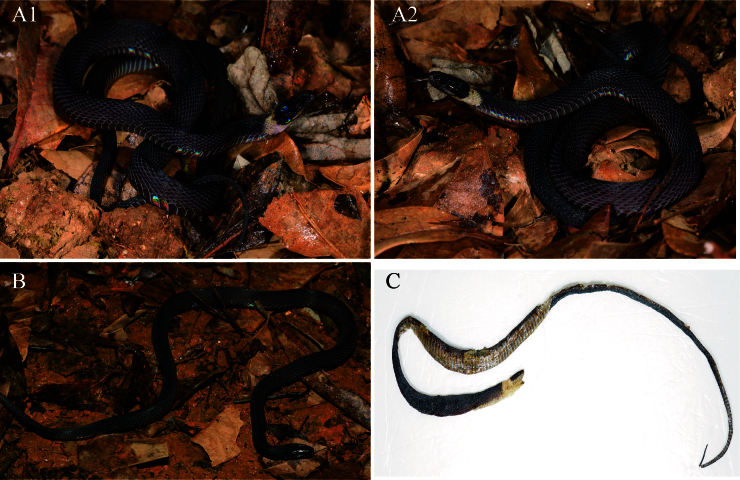
*Achalinus hunanensis *in life, **A1, A2** GXNU20240905009, adult female; **B** GXNU2024102112, adult male; **C** GXNU2024090120, adult female; photos by Li Ding.

In life, specimens display iridescent, uniformly brownish-black dorsal colouration, with a distinctive occipital bright patch extending to the head and abdomen in the two females from Guangxi (absent in males). The first chin shield pair black and second pair brown in the two females (uniformly brownish-black in males). The ventral surface is lighter brownish-black than the dorsum, with the supralabials and infralabials the darker dorsal colouration. Head scales are similar to the body colour. Ventral scale margins are greyish-white. Eyes are completely black with a vertically subelliptic pupil (Fig. 2).

**Figure 3.**
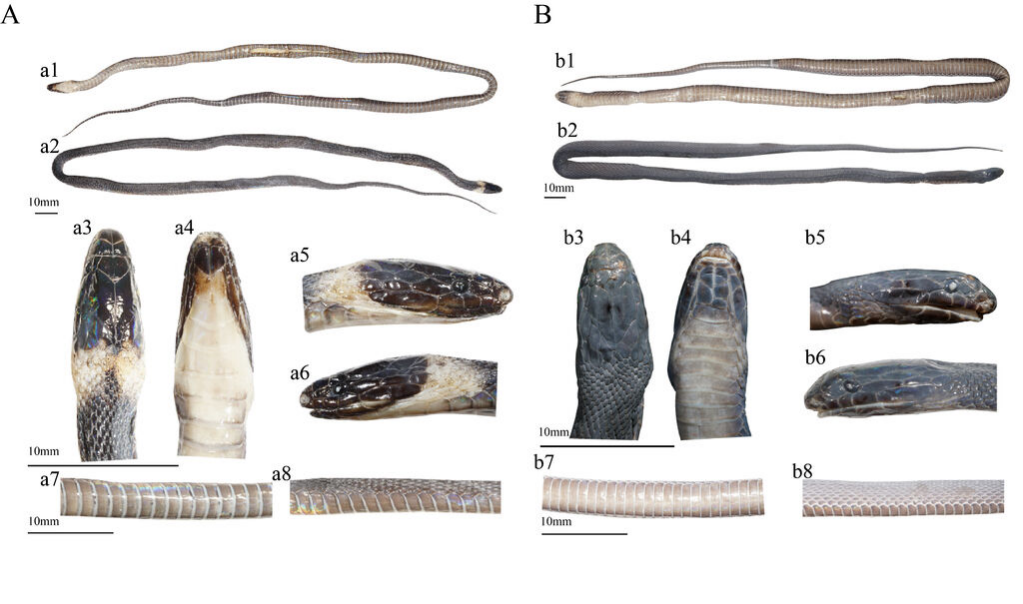
*Achalinus hunanensis*. **A** GXNU20240905009, female; **B** GXNU2024102112, male, preserved specimens: 1, ventral view; 2, dorsal view; 3, dorsal head; 4, ventral head; 5, right view of head; 6, left view of head; 7, ventral middle body; 8, lateral middle body. Photos by Yan-Yuan Xie.

In preserved specimens, the dorsum remains taupe and the venter fades to pale brownish-grey, darkening towards the caudal tip (Fig. 3).

#### Distribution

**Figure 4.**
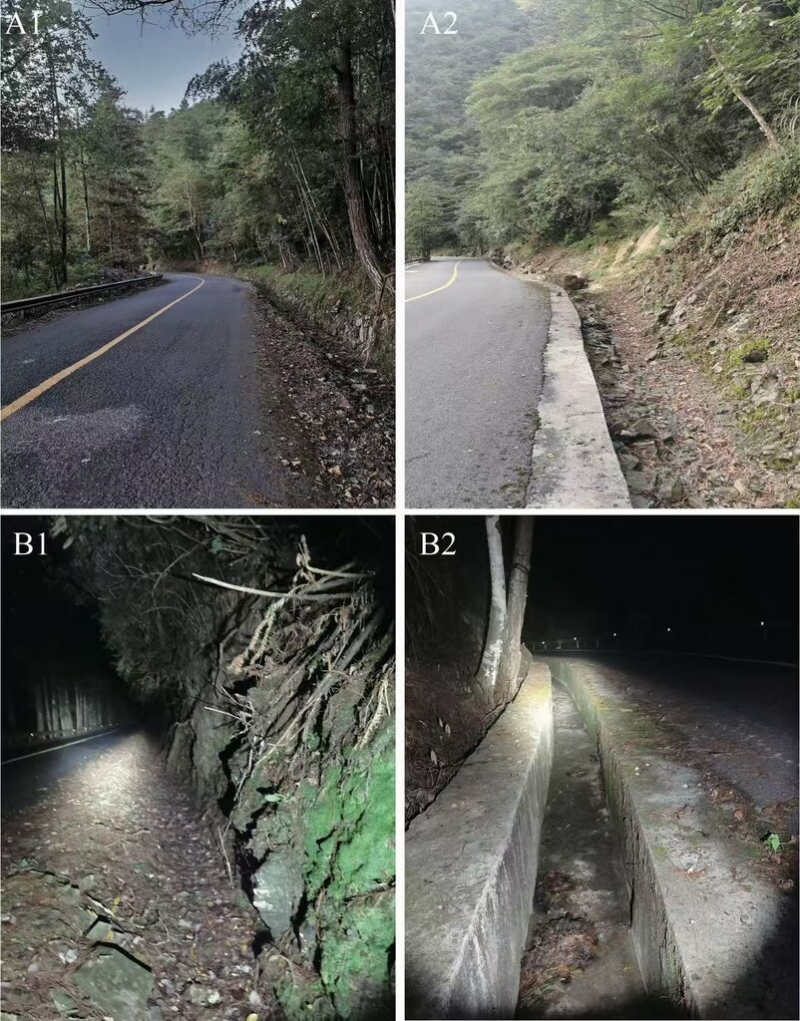
Habitats of *Achalinus hunanensisis *in Jiuwanshan National Nature Reserve, Guangxi; evening (A) and night (B); photos by Li Ding.

*Achalinus hunanensisis* is known from the following localities: Guangxi (Rongshui Miao Autonomous County), Hunan Province (Anhua County, Hecheng District, Ningxiang County) and Guizhou Province (Dushan County) (Fig. 1). The surrounding habitat, typical for all Achalinus hunanensis, comprises montane evergreen broadleaf forest (elevation 891-1281 m) with the ground covered by leaf litter (Fig. 4).

#### Notes

In the present study, the female specimens exhibit a broad white neck band that is widening towards the venter and extends to the chin region. This feature is unique to females in this study and has not been reported in previous specimens. Additionally, differences were observed in specific morphometric and scalation characteristics. For instance, the examined male specimen has a relatively larger body size (total length: 456 mm vs. 262-379 mm; tail length: 106 mm vs. 58-91 mm in previously reported males). The LSBI/LSBP ratio in this study was also lower (1.68-1.85 vs. 2.00-2.11 in previous studies). The male specimen in our study exhibits an unusual chin shield configuration: five scales in total (left side with two scales, right side with three scales), featuring normally developed first pairs, while the second pair on the right side are divided into two smaller scales. The main morphological characteristics of *A. hunanensis *are summarised in Table 2.

#### Revised diagnosis and variation of the species

(1) Dorsal scales strongly keeled, 23 rows throughout; (2) tail relatively short, TAL/TL ratio 0.22-0.24 in males and 0.17-0.19 in females; (3) maxillary teeth 23; (4) length of suture between internasals substantially longer than that between prefrontals, LSBI/LSBP ratio 1.68-2.11; (5) one loreal, subrectangular; (6) six supralabials, the fourth and fifth in contact with the eye; (7) the two anterior temporals in contact with eye; (8) ventrals 163-168 in males and 169-173 in females; (9) subcaudals 69-72 in males and 53-61 in females, not paired; (10) Some female specimens exhibit a distinctive occipital bright patch extending to the head and abdomen.

## Analysis

**Figure 5.**
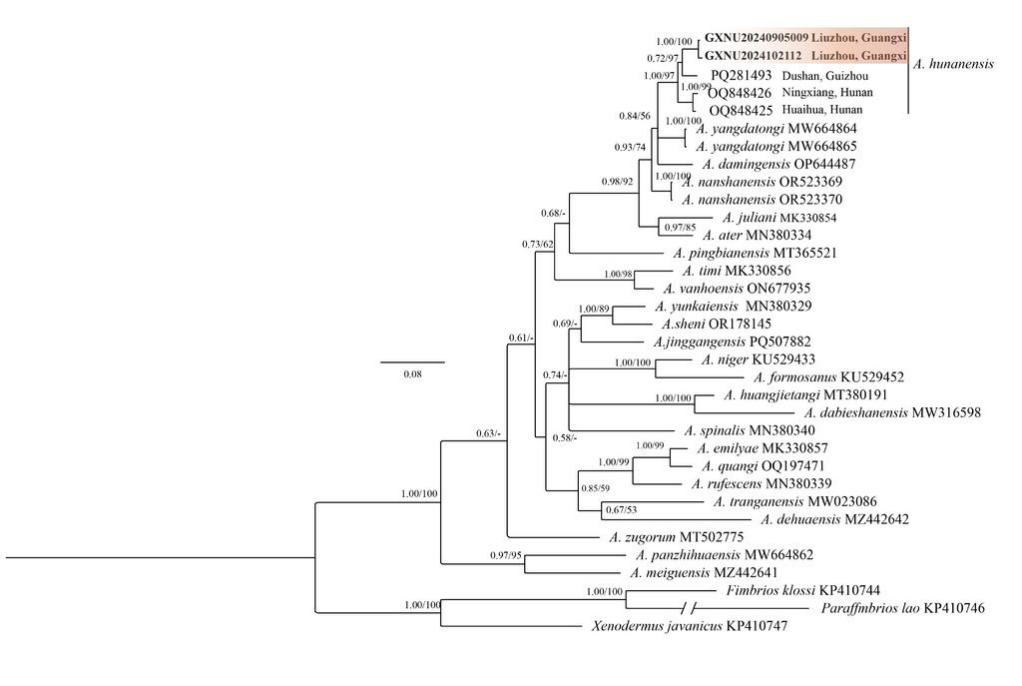
Phylogenetic relationships of *Achalinus* species, based on COI. Bayesian posterior probabilities (BPP) and Ultrafast bootstrap approximation (UFB) were denoted beside each node (those lower than 0.5/50 were denoted as "-").

The DNA dataset includes 35 COI gene sequences, spanning 640 base pairs. Both ML and BI analyses produced nearly identical topologies, as shown in Fig. 5, with corresponding genetic distances provided in Table 3. Phylogenetic reconstructions, based on the COI gene, were consistent with previous studies. All specimens collected in this study formed a single monophyletic group with strong support (BPP 1.00/UFB 100). The specimens GXNU20240905009 and GXNU2024102112 initially clustered with Guizhou Province populations (SH 0.65/UFB 84) and later formed a well-supported clade with the type series (SH 1.00/UFB 98), exhibiting low intraspecific genetic divergence (0.47-3.9%) (Table 3). Molecular and morphological evidence confirms the Rongshui County (Guangxi) specimens as *A. hunanensis*.

## Discussion

Within the family Xenodermatidae, occipital bright patches are present in both the genera* Achalinus *and *Parafimbrios*, though the colouration patterns differ: *Parafimbrios lao *exhibits white patches, whereas *A. nanshanensis, A. damingensis* and *A. jianghuaensis *display bright yellow patches. Our initial taxonomic assignment of these three specimens to *P. lao* was primarily based on the presence of a similarly extensive occipital white patch extending anteriorly to the head and posteriorly along the abdomen in the female specimen. In contrast, this patch is absent in the female specimen from Guizhou. However, subsequent detailed morphological examination revealed that these specimens lack the most diagnostic features of *P. lao *— specifically, the raised and erected edges on the rostral scale and on the first four supralabial and infralabial scales. Furthermore, compared with *P. lao*, the three specimens exhibit markedly lower counts in several key pholidosis characters: dorsal scale rows 23-23-23 (vs. (27-29）-（25-27）-（23-25）), supralabials 6 (vs. 7–8) and infralabials 5 (vs. 7–8). (Teynie et al. 2015, Ziegler et al. 2018, Cai et al. 2020, Yang et al. 2023).

The extreme scarcity of research specimens presents particular challenges for studies on *Achalinus*, as exemplified by our population of *A. hunanensis* from Guangxi, which exhibits notable morphological divergence from previous records. Most strikingly, amongst the three specimens we collected, two females display a distinct occipital bright patch extending to the head and abdomen — a diagnostic feature entirely absent in males. Specifically, the three male specimens of *A. hunanensis* from its type locality in Hunan and the single female specimen recorded in Guizhou lack this feature. In contrast, while such a patch has been documented in previously described species like* A. nanshanensis, A. damingensis *and *A. jianghuaensis*, all reported specimens of these species are males. In the present study, amongst the three collected specimens, only the two females possess this patch, marking the first record of such a feature in female individuals within the genus *Achalinus*. Furthermore, as this patch differs from that of a female *A. hunanensis* from Guizhou, further morphological study of these specimens is essential to refine the species description. In addition, compared with *A. nanshanensis*, the three specimens exhibit markedly lower counts in several key pholidotic characters: dorsal scale rows 23-23-23 (vs. (23-25)-(23-25)-(23-25)) and infralabials 5 (vs. 6). Our specimens also differ from *A. jianghuaensis* in temporal scale arrangement (TEMP 2+2+3 vs. 2+2+4). Collectively, these morphological differences clearly distinguish our specimens from both *A. jianghuaensis* and *A. nanshanensis *(Li et al. 2024, Liang et al. 2024).

The odd-scaled snakes inhabit humid forest-edge microhabitats and natural clearings with abundant humus deposits (Zhao 2006, Yang et al. 2023). Their secretive habits, small size and cryptic colouration make them highly elusive in nature. The bright head-neck patch in *Achalinus* likely serves as both a dynamic warning and static camouflage. During movement, it acts as a conspicuous, high-contrast signal to startle or deter predators. When stationary, it mimics forest-floor light, disrupting its outline to enhance concealment — a multifunctional adaptation for its leaf-litter habitat（STUART-FOX et al. 2006, Cyriac and Kodandaramaiah 2019, Cyriac and Kodandaramaiah 2020）. Previously, *A. hunanensis* was only recorded from its type locality in Hunan and from Guizhou Province, with the holotype and paratype collected at 880 m and 1,200 m, respectively (Ma et al. 2023b). Specimens from Jiuwanshan National Nature Reserve, Guangxi, at 891-1281 m elevation, extend the species' geographic range within its known altitudinal range.

Recent studies combining morphology and molecular analyses showing that the genus' diversity is still underestimated (Xu et al. (2024), Huang et al. (2024), Li et al. (2024), Xu et al. (2025)). Targeted surveys are needed to fully understand the true diversity and distribution patterns of this genus.

## Supplementary Material

XML Treatment for Achalinus hunanensis

## Figures and Tables

**Table 1.** Localities, voucher information, GenBank accession numbers, and references for the samples used in this study.

**No.**	**Species**	**Voucher**	**Locality**	**GenBank**	**References**
1	*A. hunanensis*	GXNU20240905009	Rongshui, Liuzhou, Guangxi, China	PX048858	This study
2	*A. hunanensis*	GXNU2024102112	Rongshui, Liuzhou, Guangxi, China	PX048859	This study
3	*A. hunanensis*	CIB119040	Ningxiang, Hunan, China	OQ848426	Ma et al. (2023b)
(4	*A. hunanensis*	CIB119039	Huaihua, Hunan, China	OQ848425	Ma et al. (2023b)
5	*A. hunanensis*	QHU 2024030	Dushan, Guizhou, China	PQ281493	Xu et al. (2024)
6	*A. niger*	RN0667	Taiwan, China	KU529433	Unpublished
7	*A. formosanus*	RN2002	Taiwan, China	KU529452	Unpublished
8	*A. juliani*	IEBRA.2018.8	HaLang, Cao Bang, Vietnam	MK330854	Ziegler et al. (2019)
9	*A. timi*	IEBRA.2018.10	ThuanChau, Son La, Vietnam	MK330856	Ziegler et al. (2019)
10	*A. emilyae*	IEBR4465	HoanhBo, Quang Ninh, Vietnam	MK330857	Ziegler et al. (2019)
11	*A. yunkaiensis*	SYSr001443	Dawuling Forestry Station, Guangdong, China	MN380329	Wang et al. (2019)
12	*A. ater*	SYSr00852	Huaping Nature Reserve, Guangxi, China	MN380334	Wang et al. (2019)
13	*A. rufescens*	SYSr001866	Hongkong, China	MN380339	Wang et al. (2019)
14	*A. spinalis*	SYSr001327	Badagong Mountains, Hunan, China	MN380340	Wang et al. (2019)
15	*A. pingbianensis*	YBU18273	Honghe, Yunnan, China	MT365521	Li et al. (2020)
16	*A. huangjietangi*	HSR18030	Huangshan, Anhui, China	MT380191	Huang et al. (2021)
17	*A. zugorum*	IEBR4698	Bac Me, Ha Giang, Vietnam	MT502775	Miller et al.(2020)
18	*A. tranganensis*	VNUFR.2018.21	NinhBinh, Vietnam	MW023086	Luu et al. (2020)
19	*A. dabieshanensis*	AHU2018EE0710	Fuziling Provincial Reserve, Anhui, China	MW316598	Zhang et al. (2023)
20	*A. panzhihuaensis*	KIZ040189	Yanbian, Sichuan, China	MW664862	Hou et al. (2021)
21	*A. yangdatongi*	YPX51447	Xichou county,Yunnan,China	MW664864	Xu et al. (2023)
22	*A. yangdatongi*	KIZ034327	Wenshan Nature Reserve，Yunnan，China	MW664865	Hou et al. (2021)
23	*A. meiguensis*	GP835	Mianyang, Sichuan, China	MZ442641	Li et al. (2021)
24	*A. dehuaensis*	YBU13013	Dehua, Fujian, China	MZ442642	Li et al. (2021)
25	*A. vanhoensis*	VNUFR.2019.13	VanHo, Son La, Vietnam	ON677935	Ha et al. (2022)
26	*A. damingensis*	ANU20220009	Shanglin, Nanning, Guangxi, China	OP644487	Yang et al. (2023)
27	*A. quangi*	sp4	Northern Vietnam	OQ197471	Pham et al. (2023)
28	*A.sheni*	ANU20230012	Lianyuan,Hunan,China	OR178145	Ma et al. (2023c)
29	*A. nanshanensis*	HNNU230902	Nanshan National Park, Hunan, China	OR523369	Li et al. (2024)
30	*A. nanshanensis*	HNNU230903	Nanshan National Park, Hunan, China	OR523370	Li et al. (2024)
31	*A.jinggangensis*	QHU2023011	Fujian,China	PQ507882	Xu et al. (2024)
32	*A. jianghuaensis*	JSUWT005	Jianghua, Hunan, China	OR161056	Huang et al. (2024)
33	*Fimbrios klossi*	IEBR3275	Quang Ngai, Vietnam	KP410744	Teynie et al. (2015)
34	*Paraffmbrios lao*	MNHN2013.1002	Louangphabang, Laos	KP410746	Teynie et al. (2015)
35	*Xenodermus javanicus*	--	Sumatera Barat,Indonesia	KP410747	Teynie et al. (2015)

**Table 2.** Morphological variation of *Achalinus hunanensis *obtained from specimens examined in this study and Ma et al. (2023b) and Xu et al. 2025. *= left two scales, right three scales and the second pair on the right side divided into two smaller scales. Abbreviations as defined in 'Morphological examination'.

Voucher number	GXNU 2024102112	GXNU 20240905009	GXNU 2024090120	QHU 2024030	CIB 20160503	CIB 119039	CIB 119040
Location	Liuzhou, Guangxi	Liuzhou, Guangxi	Liuzhou, Guangxi	Dushan, Guizhou	Anhua, Hunan	Hecheng, Hunan	Ningxiang, Hunan
Sex	♂	♀	♀	♀	♂	♂	♂
Presence or absence of an occipital patch	absence	presence	presence	absence	absence	absence	absence
SVL	350	329	290	355	288	255	204
TL	456	401	358	428	379	329	262
TAL	106	72	68	73	91	74	58
TAL/TL	0.23	0.18	0.19	0.17	0.24	0.23	0.22
HL	14.27	12.18	-	12.5	12.7	7.9	6.3
HW	7.67	5.4	-	5.9	4.6	4.8	3.4
SL	3+2+1	3+2+1	-	3+2+1	3+2+1	3+2+1	3+2+1
IL	5/5	5/5	-	6/6	5/5	5/6	5/5
Chins	2*	2	-	2	2	2	2
IL-1stChin	3/3	3/3	-	3/3	3/3	3/4	3/3
Lor	1	1	-	1	1	1	1
LorH	1.35	1.06	-	1.0	1.2	1.0	0.9
LorL	1.78	1.54	-	1.6	1.7	1.5	1.5
LorH/LorL	0.814	0.69	-	0.63	0.71	0.67	0.60
LSBI	2.5	1.43	1.63	1.9	1.8	1.78	1.52
LSBP	1.35	0.85	0.93	0.9	0.9	0.88	0.76
LSBI/LSBP	1.85	1.68	1.75	2.11	2.00	2.02	2.00
LSBI vs.LSBP	>	>	>	>	>	>	>
ED	1.41	0.96	-	1.2	1.1	1.4	1.4
TEMP	2+2+3/2+2+3	2+2+3/2+2+3	-	2+2+3/2+2+3	2+3+4/2+3+4	2+2+4/2+2+4	2+2+4/2+2+4
aTEMP-Eye	2/2	2/2	-	2/2	2/2	2/2	2/2
SPO	1	1	-	1	1	1	1
DSR	23-23-23	23-23-23	-	23-23-23	23-23-23	23-23-23	23-23-23
VS	163	173	-	169	168	163	165
CP	1	1	1	1	1	1	1
SC	71	61	-	53	69	69	72

**Table 3.** Uncorrected p-distances (%) amongst the *Achalinus* species, based on partial mitochondrial COI gene for species compared in this study.

No.	Species	1-2	3-5	6	7	8	9	10	11	12	13	14	15	16
1-2	*A. hunanensis *(this study)	0.47												
3-5	*A. hunanensis*	2.82-3.90	0.52-3.38											
6	*A. niger*	17.14-17.34	14.80-15.71											
7	*A. formosanus*	16.88-17.09	15.58-16.44	9.73										
8	*A. juliani*	10.31-10.67	9.48-9.57	14.00	14.49									
9	*A. timi*	16.32-16.72	13.31-14.29	13.62	15.89	15.67								
10	*A. emilyae*	14.70-15.09	14.21-14.92	14.05	16.03	13.64	14.73							
11	*A. yunkaiensis*	14.20-14.59	12.96-13.35	13.55	13.85	13.97	15.94	14.90						
12	*A. ater*	8.56-8.74	7.47-7.69	15.22	16.18	7.44	15.02	13.14	14.30					
13	*A. rufescens*	13.87	13.60-13.67	14.20	16.00	13.84	15.76	8.55	15.04	14.31				
14	*A. spinalis*	16.74-16.94	15.60-16.53	15.26	15.85	15.84	16.18	15.91	13.33	17.45	14.62			
15	*A. pingbianensis*	13.25-13.64	12.19-13.04	13.14	16.66	13.53	13.65	14.64	12.88	13.08	14.53	14.92		
16	*A. huangjietangi*	20.89-21.13	18.75-19.55	16.25	18.67	16.79	16.66	16.72	13.97	17.26	16.30	15.11	14.58	
17	*A. zugorum*	14.49-14.88	13.03-13.61	15.43	15.60	15.05	15.41	14.17	12.12	14.88	15.17	15.08	12.15	16.32
18	*A. tranganensis*	16.43-16.82	15.43-15.97	16.53	19.63	14.83	15.43	12.35	15.18	14.29	12.71	16.65	14.88	15.25
19	*A. dabieshanensis*	19.08	19.39-20.10	18.40	22.76	18.44	19.02	20.96	16.96	16.91	19.97	19.26	17.53	9.50
20	*A. panzhihuaensis*	19.41	19.00-19.56	16.61	18.84	18.03	17.87	19.44	18.02	18.96	18.66	18.40	17.21	17.52
21-22	*A. yangdatongi*	5.90	5.29-5.70	15.45	16.52	7.73	14.76	14.41	13.33	6.48	12.85	16.11	12.50	16.71
23	*A. meiguensis*	19.64	18.86-19.14	15.73	18.04	19.60	18.09	17.80	18.05	17.84	20.36	18.49	19.55	17.50
24	*A. dehuaensis*	18.29-18.48	17.1-18.40	18.49	18.31	17.51	18.76	17.97	16.86	19.27	16.17	16.24	17.07	19.41
25	*A. vanhoensis*	15.10-15.31	12.74-12.75	14.30	15.98	15.31	5.41	13.90	15.31	14.75	15.57	14.47	11.81	16.60
26	*A. damingensis*	6.55-6.90	6.35-6.57	16.16	17.11	9.07	15.14	14.67	13.92	8.72	15.61	17.20	12.50	18.92
27	*A. quangi*	15.05-15.63	13.80-14.93	13.45	16.17	14.26	15.33	4.03	15.49	13.17	8.36	15.88	15.84	16.91
28	*A. jianghuaensis*	5.45	5.48-6.29	16.81	16.48	8.39	15.05	14.68	13.87	7.08	13.81	16.14	13.47	19.06
29	*A.sheni*	14.27-14.47	13.46-14.19	14.17	14.82	15.67	15.70	15.11	6.58	14.45	14.90	12.57	12.79	15.34
30-31	*A. nanshanensis*	5.51	4.85-5.40	14.94	17.09	8.33	15.32	14.44	13.70	7.08	13.41	15.71	12.83	19.29
32	*A.jinggangensis*	13.83-14.22	12.99-13.75	11.79	13.24	13.43	15.11	14.25	9.23	13.62	13.19	11.18	12.54	12.14
No.	Species	17	18	19	20	21-22	23	24	25	26	27	28	29	30-31
18	*A. tranganensis*	13.53												
19	*A. dabieshanensis*	17.59	17.58											
20	*A. panzhihuaensis*	17.56	18.98	19.32										
21-22	*A. yangdatongi*	13.73	14.43	19.36	18.02									
23	*A. meiguensis*	17.04	18.83	20.69	12.75	20.11								
24	*A. dehuaensis*	16.81	15.85	21.82	17.64	16.00	21.13							
25	*A. vanhoensis*	13.61	15.07	17.71	17.85	12.48	17.83	18.58						
26	*A. damingensis*	14.45	15.83	18.31	17.92	5.89	19.50	18.55	14.04					
27	*A. quangi*	14.25	13.70	21.43	19.96	14.17	17.59	17.65	14.07	14.86				
28	*A. jianghuaensis*	14.37	14.81	19.87	18.48	4.91	20.36	16.90	13.97	5.63	14.87			
29	*A.sheni*	11.94	14.94	18.26	16.64	15.49	15.81	15.29	15.94	15.64	15.84	13.97		
30-31	*A. nanshanensis*	14.42	14.80	18.52	17.94	4.51	20.78	16.66	13.81	5.49	14.44	5.09	15.21	
32	*A.jinggangensis*	12.95	15.54	16.50	17.86	12.99	14.44	16.22	13.57	13.58	14.43	13.86	9.60	12.97
